# An Electronic System for the Contactless Reading of ECG Signals

**DOI:** 10.3390/s17112474

**Published:** 2017-10-28

**Authors:** Francesca Romana Parente, Marco Santonico, Alessandro Zompanti, Mario Benassai, Giuseppe Ferri, Arnaldo D’Amico, Giorgio Pennazza

**Affiliations:** 1 Department of Industrial and Information Engineering and Economics, University of L’Aquila, 67100 L’Aquila, Italy; francescaromana.parente@graduate.univaq.it (F.R.P.); giuseppe.ferri@univaq.it (G.F.); 2 Unit of Electronics for Sensor Systems, Department of Engineering Campus Bio-Medico University of Rome, 00128 Rome, Italy; m.santonico@unicampus.it (M.S.); a.zompanti@unicampus.it (A.Z.); 3 ALTEC S.p.A., Aerospace Logistics and Technology Engineering Company, 10146 Torino, Italy; mario.benassai@altecspace.it; 4 Department of Electronic Engineering, University of Rome Tor Vergata, 00133 Rome, Italy; damico@eln.uniroma2.it

**Keywords:** capacitive sensors, ECG, contactless, biomedical electronics, wearable

## Abstract

The aim of this work is the development of a contactless capacitive sensory system for the detection of (Electrocardiographic) ECG-like signals. The acquisition approach is based on a capacitive coupling with the patient body performed by electrodes integrated in a front-end circuit. The proposed system is able to detect changes in the electric charge related to the heart activity. Due to the target signal weakness and to the presence of other undesired signals, suitable amplification stages and analogue filters are required. Simulated results allowed us to evaluate the effectiveness of the approach, whereas experimental measurements, recorded without contact to the skin, have validated the practical effectiveness of the proposed architecture. The system operates with a supply voltage of ±9 V with an overall power consumption of about 10 mW. The analogue output of the electronic interface is connected to an ATmega328 microcontroller implementing the A/D conversion and the data acquisition. The collected data can be displayed on any multimedia support for real-time tracking applications.

## 1. Introduction

In recent years, there has been a growing interest in the detection of signals from the human body [1,2,3,4]. Typically, these measurements are performed by the use of Ag/AgCl electrodes with conductive gels in direct contact with the skin in order to transduce the body surface ion current into an electron current [5,6,7]. These bioelectric signals represent the result of the synchronous electrochemical activity of the nervous tissue cells. Hence, a signal picked up from the human body consists of an analogue signal, which is characterized by low intensity, low frequency and narrow bandwidth. In particular, traditional electrocardiography, ECG, is able to record those potentials on the body surface, directly reflecting the electrical cardiac activity with an amplitude of few mV with frequencies in the range of 0.01–150 Hz [8]. In addition, in the electrocardiographic acquisition process, it is necessary to filter out some undesirable signals, such as, for example, those caused by the power-line supply (50 Hz in Europe, 60 Hz in the USA) or those coming from other bioelectrical sources [9].

Typically, the use of contact standard electrodes requires a proper preparation of the body surface due to the presence of body hair, abrasions or dirt [10]. Moreover, prolonged use of the gel can cause irritation, as well as the loss of its conductive capacity, caused by a drying out over time [11]. In order to overcome these drawbacks, dry contact electrodes have been widely investigated for long-term heart monitoring applications [12,13]. In some applications, these transducers have shown better performance with respect to traditional wet gel electrodes in terms of electrode–skin interface impedance, signal intensity and size [14].

In the last years, non-contact electrodes have been considered as an alternative option with respect to contact electrodes [15,16]. In particular, in the literature, capacitive sensors performing a coupling with a human body have been explored [17,18,19,20,21]: whilst they still have not replaced traditional wet electrodes, they have shown satisfactory results in particular applications, such as in the automotive environment [20] or in wearable health devices [21]. Having an electrode without direct contact with the skin offer several benefits in long-term monitoring applications, such as stability over time and having the capacitive sensor surface electrically insulated [22]. In addition, a contactless monitoring system may be more comfortable for the involved subject, as it is user-friendly, not invasive at all and it does not require the presence of qualified medical staff.

In this paper, a complete system for the contactless real-time monitoring of ECG signal is presented. It exploits electrodes performing a capacitive coupling with the patient body that through a dedicated front-end circuit are able to detect electrical charge variations related to the heart activity. This work represents an optimized version of an already-presented system that shows only simulated results for a preliminary version, employing one single capacitive electrode [23]. In this study, two leading electrodes and one more as reference (that has to be placed far from the cardiac source) were included and the front-end was been completely redesigned.

The promising results obtained here pave the way to wearable solutions for an ECG contactless device via a suitable integration of the system described in the paper. The active filtering effectively supports the extraction of a clear signal at the frequency of interest, as shown by the experimental results reported here, which validate the practical effectiveness of the proposed approach.

## 2. The Proposed System

**Figure 1:**
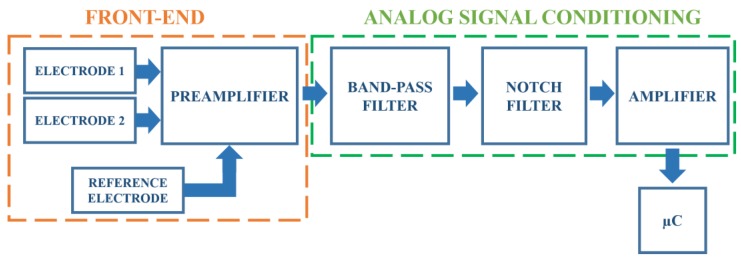
The proposed system block scheme.

In this section, the details of the designed system are reported. The block scheme reported in Figure 1 summarizes the main stages. The detection of the heart activity, through the contactless electrodes, occurs at the front-end portion (that includes the electrodes and a preamplifier), whose output signal is then fed to an analogue signal conditioning circuit formed by filters and by an amplifier. The amplified output signal is picked up by a microcontroller (μC) dealing with the analogue-to-digital conversion and the digital data storage.

In order to evaluate the circuit limits, as well as to optimize its performance, simulations of the analogue electronic filter stages were performed by using National Instruments NI Multisim software. As an operational amplifier, LF411 was employed [24]. All the active elements in the electronic interface were supplied at ±9 V.

### 2.1. The Front-End Block

**Figure 2:**
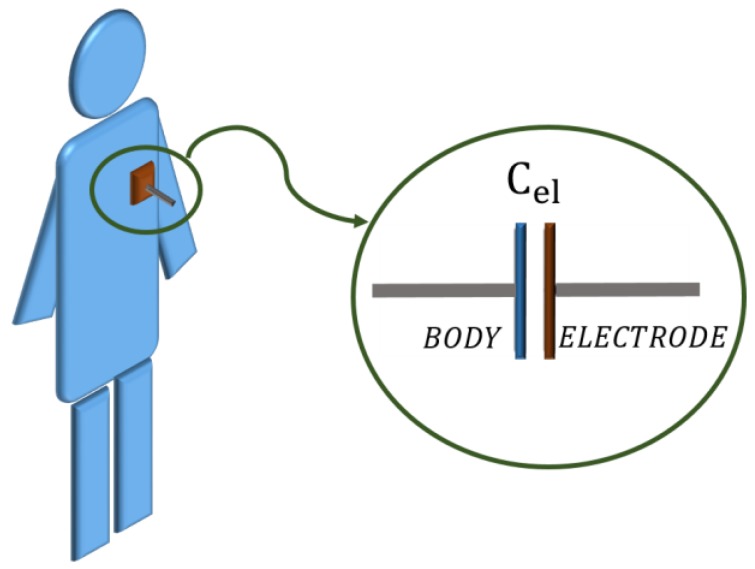
Electrode positioning on the human body.

ECG monitoring through capacitive measurement requires a dedicated electrode. In this work, an electrode made up of a single conductive (copper) layer was designed. This electrode, when brought close to the subject’s chest (see Figure 2), can be modelled through a capacitor *C_el_* whose value, according to the parallel plate capacitor theory, depends on the distance, on the size and on the dielectric material between the two conductors [25].

Having considered a single layer cotton T-shirt (dielectric constant: *ε_cotton_* = 1.4) with a thickness of roughly 0.5 mm and a fixed size for the electrodes of about 65 cm^2^, an equivalent capacitance value of about 150 pF was estimated. A high valued resistor (few GΩ), connected to ground, in series with this capacitor, forms a passive high-pass cell at very low frequencies (of about 1 Hz).

A variation in both the material (*ε_r_*) and the thickness (*t*) of the t-shirt results in a modification of the cutoff frequency. The dependence of the overall transfer function on these two parameters has been verified by simulation—*ε_r_* =1.3 ÷ 1.6, *t* = 1, 0.75, 0.50, 0.25 mm—these ranges have been found in the literature [18,26,27]. At low frequency, the range of interest of the ECG signal, both the material and the thickness influence the transfer function. It is worth remarking that, although the thickness effect seems to be more significant than the material, they both do not prevent effective signal capturing.

**Figure 3:**
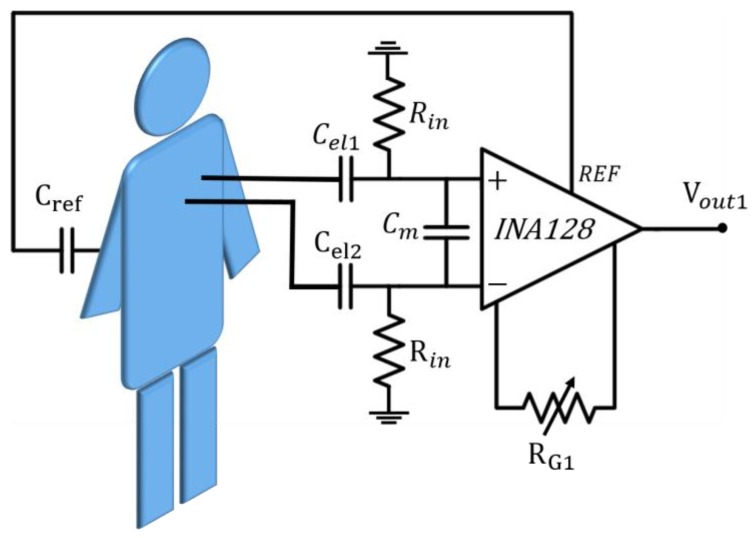
The front-end block.

The high-pass filter is present on both of the two leading electrodes, as shown in Figure 3, which also includes the first analogue front-end amplifier.

The signal associated with cardiac activity typically shows very low voltage levels in a standard contact acquisition and is also lower in this approach, therefore the presence of a preamplifier is mandatory. In order to match the high resistor, a high input impedance instrumentation amplifier is included (INA128 [28]). This amplifier shows a high common mode rejection ratio (CMRR). A single external resistor *R_G_*_1_, whose default chosen resistance value is 12 kΩ, sets the amplifier gain to G1 ≈ 5, i.e., about 14 dB. Due to both the interpersonal variability and the measurement conditions, this value might be unsuitable in some cases. Hence, we included a trimmer (*R_G_*_1_), which allows adjustment of the gain value. In addition, a capacitor between the input terminals, *C_m_*, helps to reduce the magnetic coupling noise.

### 2.2. The Band-Pass Filter

**Figure 4:**
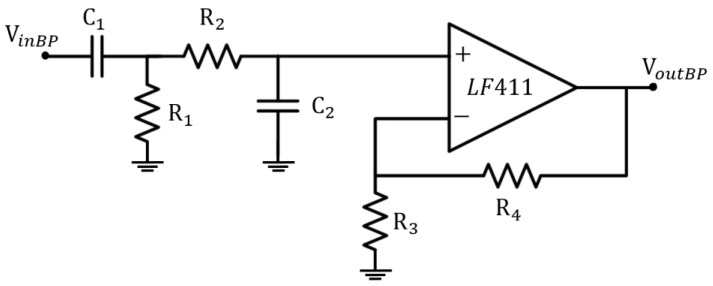
Active band-pass filter schematic circuit.

**Figure 5:**
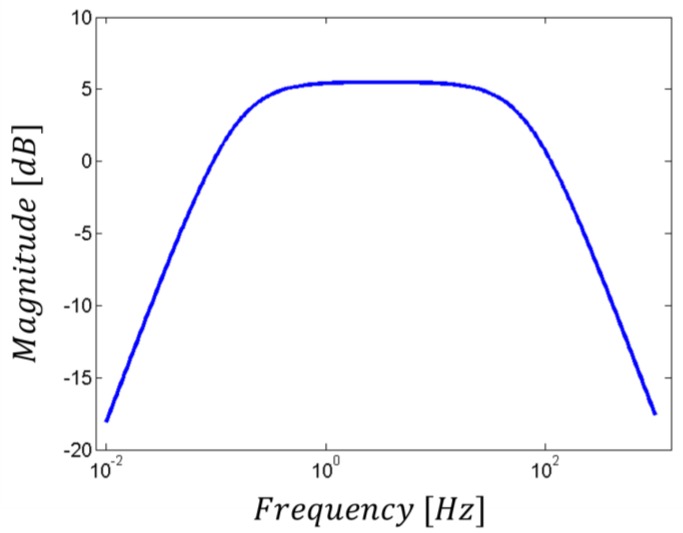
Active band-pass filter transfer function.

The first filtering stage is represented by an active band-pass filter (see Figure 4), which limits the bandwidth to the frequencies of interest in the ECG signal [9]. This design choice does not affect the acquisition process (while, indeed, it helps in the highlighting of the information of interest), since the most significant part of the ECG signal lies below 30 Hz, even if it can be up to 150 Hz [8]. Moreover, its cut-off at low frequencies (around 0.2 Hz) reduces the baseline drift due to respiration. Furthermore, the resistors in the loop circuit allow the gain control of the signal in the bandwidth, which has an amplification factor of about 5 dB, as shown in Figure 5.

### 2.3. The Notch Filter

**Figure 6:**
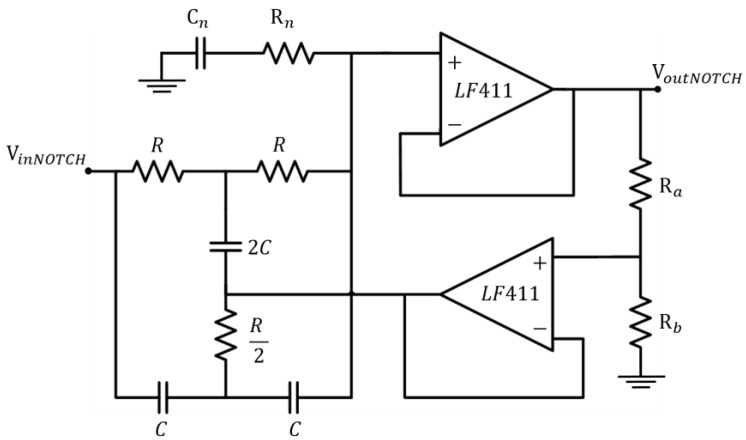
Modified Twin-T notch filter schematic circuit.

A second-order Twin-T notch filter centred on the frequency of 50 Hz obtains the reduction of the power supply interference. In particular, in order to increase the attenuation feature, the standard Twin-T configuration was modified by the addition of extra passive components, *C_n_* and *R_n_* (see Figure 6).

The values of these two additional components can be set according to (1) and (2):

*C_n_* = *nC* (1)

**Figure 7:**
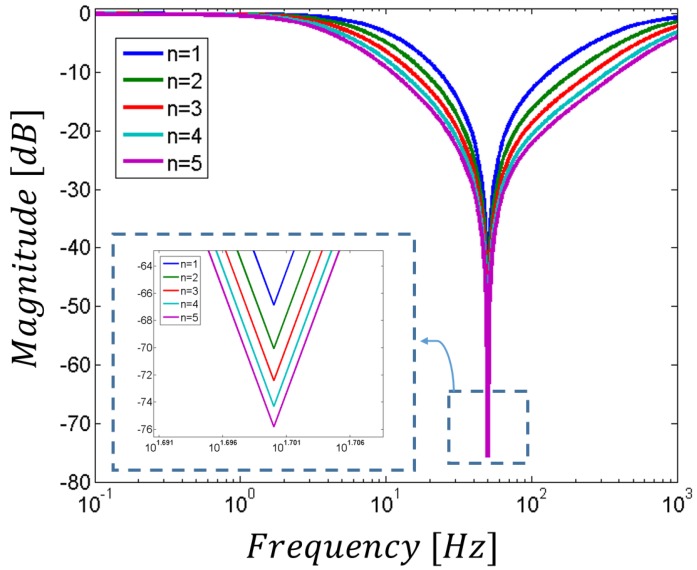
Modified notch filter transfer function and its dependence on *n*.

*R_n_* = (*R*)/2*n* (2)

where *n* is a positive integer. In this novel architecture, the cut-off frequency is still given by the components in the main passive network (i.e., it is given by *f_cutoff_*= 1/2*πRC*). However, as shown in Figure 7, the higher the n value, the higher the attenuation. On the other hand, for equal *R_a_* and *R_b_* values (that usually set the standard Q factor), the increase in the reduction of the undesired frequency results in a lower selectivity, thus in a wider stop-band. In order to limit the losses of important components of the target signal, we have chosen *n* = 1 for the notch filter included in this study.

### 2.4. The Second Amplification Stage

In the second amplification stage, a higher gain is provided in order to ensure a suitable output signal. For the considered application, an instrumentation amplifier (INA126), with a gain value of around G_2_ = 20 (26 dB), was selected. As for the first stage, a trimmer was included for gain adjusting.

### 2.5. The Whole Interface Schematic

**Figure 8:**
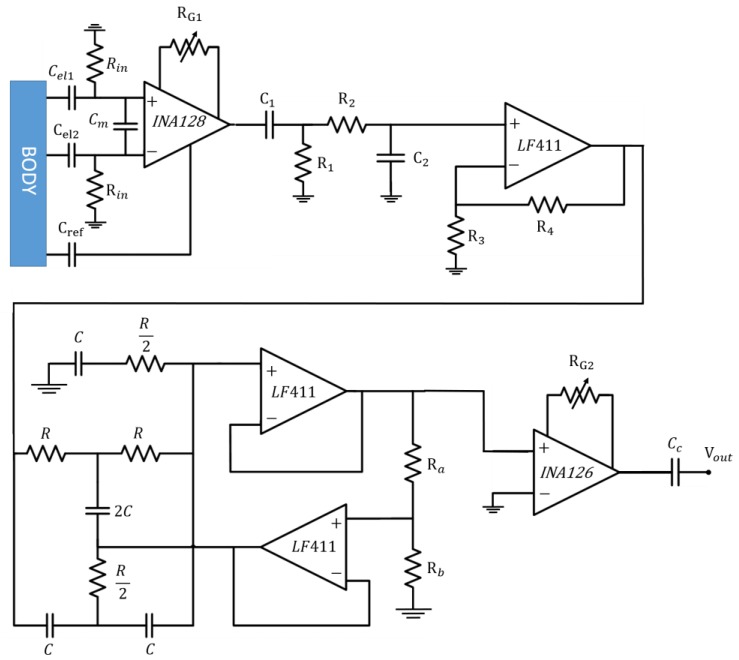
Circuit schematic of the whole analogue system.

The schematic of the proposed system, including all the analogue stages, is reported in Figure 8. The employed values of passive components are listed in Table 1. A coupling capacitor *C_c_* was placed at the end of the analogue circuit, to reject the DC components and to restore the baseline of the output signal.

**Figure 9:**
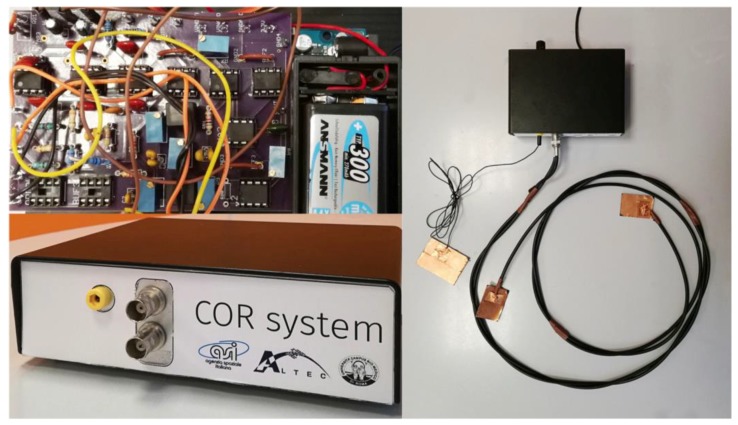
System overview (the box containing the electronic board, the electronic board, the three electrodes connected to the system).

The whole system fabricated was composed by a box containing the electronic interface described and the capacitive electrodes. All these components are reported in Figure 9.

### 2.6. Data Acquisition

A μC Atmega 328 was used for the output signal analogue-to-digital conversion. Dedicated software was implemented for data storage as well as for real-time display of the ECG signal acquired μC. In addition, digital filtering handling and further data processing were included so as to obtain a sharp signal by the action of the following digital filter: *A_n_*= *A_n_*− *E_n_*, where *E_n_*= (*αA_n_*_−1_) + (1 − *α)E_n−_*_1_, *A_n_* is the *n*-th sample, *E_n_* is the *n*-th sample, and *α* is the parameter defining the filter intensity.

## 3. Results

### 3.1. Simulated Results

**Figure 10:**
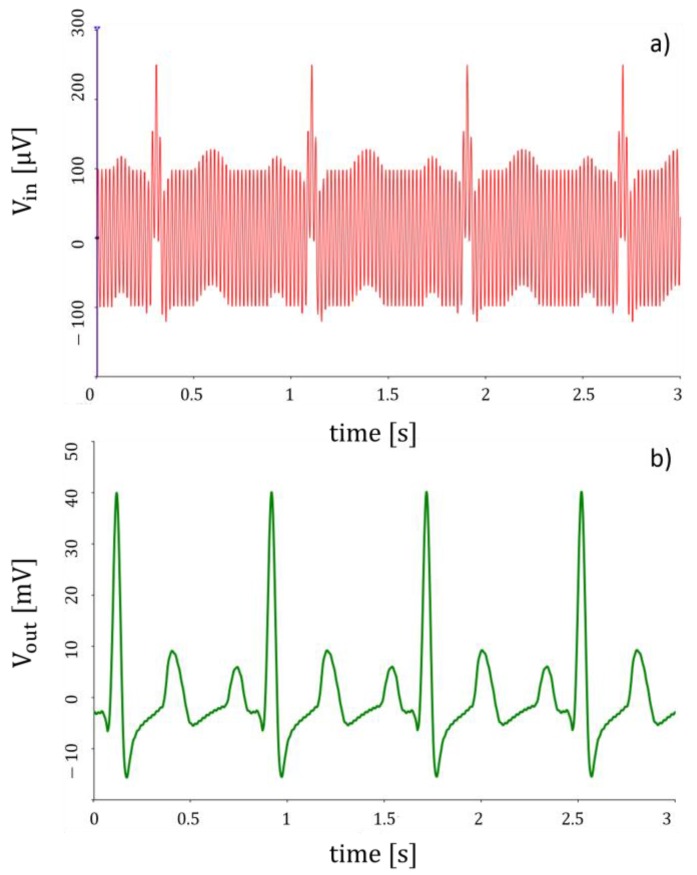
Simulation of the (**a**) input signal (**b**) output signal.

Simulations of the whole system for a typical cardiac signal were carried out by NI Multisim. Two different source generators were included in the furtherance of emulating the input signal at each leading electrode; a sinusoidal voltage generator at 50 Hz represents the power-line interference while the cardiac waveform was reproduced using a piecewise linear voltage generator. In particular, the waveform in Figure 10a represents the input signal (as a sum of the above-mentioned waveforms), whereas that one in Figure 10b is the output signal. The system provides a considerable reduction of power-line interference effects.

### 3.2. Experimental Results

**Figure 11:**
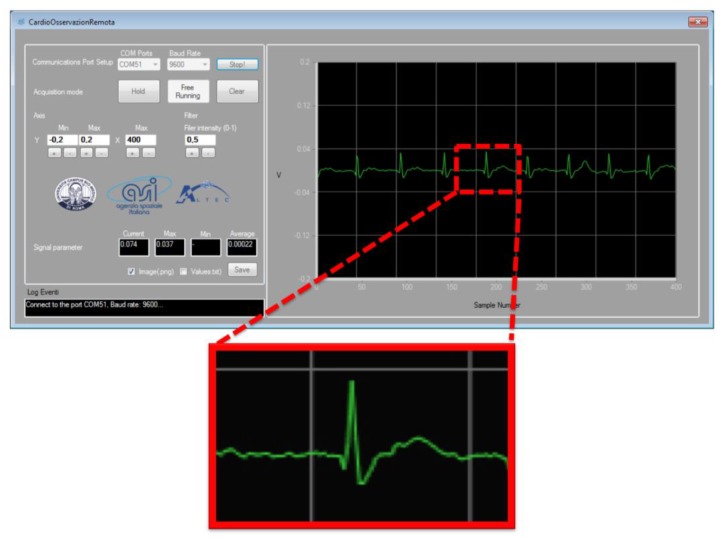
Heart signal recordings (two electrodes placed on the t-shirt of one of the ten control individuals tested). The ECG-like signal is displayed in real time on the window of the dedicated SW. The detail in the zoom put in evidence that the wave T and the complex QRST can be easily identified, while the P wave is often confused with similar artefacts.

Experimental measurements were conducted on 10 subjects wearing a single layer cotton T-shirt. Figure 11 reports a typical ECG signal recording acquired using the developed system with a sampling rate of 20 ms. For this acquisition, two electrodes were placed on the t-shirt of the control individual. A dedicated software (SW) interfaced via a dedicated Graphical User Guide (GUI), displays the ECG-like signal in real time. The wave T and the complex Q-R-S-T can be easily identified, while the P wave is not always evident. The SW GUI makes it possible to adjust the acquisition parameters while registering data, and it also allows the physician some preliminary elaborations on the signal.

## 4. Conclusions

The motivations of this research activity can be summarized by the following needs: simple procedure, portability, remote monitoring, and wearable solutions. These objectives can be achieved providing a low number of electrodes, low-power, and size reduction. In the last eight years almost 20 papers have been published on this subject; 45% of them present devices based on capacitive coupling. One third of these studies are only simulated. In this paper the complete design and development process of a device for the contactless detection of ECG-like signals is presented, starting from the design simulation and ending with the fabrication of the device and the experimental tests.

In this work, we presented a system for the contactless monitoring of human heart activity. This system has been designed to detect an ECG-like signal in non-contact mode, meaning not in direct contact with the skin, but at a certain distance, represented by the typical thickness of a basic cotton T-shirt. The electronic circuits composing the system were simulated and the prototype was tested in a real experiment on control subjects. The ECG signals registered account for an acceptable similarity with the ones obtained by the standard ECG monitoring instruments. The development of such a system opens the way for developing applications in different fields supported by distributed IoT (Internet of Things) data transfer systems such as space, industrial, and healthcare. For medical applications, the role of new wearable technologies will ensure a wider use of pre-screening, reducing the secondary effects of cardiac pathology and limiting medical care costs. On the other hand, for space applications it will allow the monitoring of the activities of astronauts without using specific and complex medical protocols. Further improvements will include the embedding of the device into some daily life households, such as in beds, chair and walls.

## Figures and Tables

**Table 1:** Component values.

Name	Value [Ω]	Name	Value [Ω]	Name	Value [F]
*R_in_*	1 G	*R_4_*	8.2 k	*C_m_*	180 p
*R_G1_*	12 k	*R*	31.82 k	*C_1_*	220 n
*R_1_*	3.3 M	*R_a_*	3.3 k	*C_2_*	100 n
*R_2_*	33 k	*R_b_*	1 k	*C*	100 n
*R_3_*	4.7 k	*R_G2_*	1.8 k	*C_c_*	15 n
